# Acral lentiginous melanoma of the foot and ankle: A case series and review of the literature

**DOI:** 10.1186/1757-1146-1-11

**Published:** 2008-09-15

**Authors:** Ivan R Bristow, Katharine Acland

**Affiliations:** 1 School of Health Sciences, University of Southampton, UK; 2 St Johns Institute of Dermatology, St Thomas' Hospital, London, UK

## Abstract

**Background:**

Acral lentiginous melanoma (ALM) is an uncommon, cutaneous malignant tumour which may arise on the foot. Its relative rarity, atypical appearance and late presentation frequently serve as poor prognostic indicators.

**Methods:**

At a tertiary skin tumour centre, a retrospective review was undertaken of all patients diagnosed with the tumour at the level of ankle or below.

**Results:**

Over a six year period, 27 cases (20 female, 7 male) were identified with positive histology confirming the disease. The age ranged from 35–96 years of age (mean 62.7 years). The majority of the cohort were white (59%) with plantar lesions (62%). 33% of patients were initially were diagnosed incorrectly. The average time taken from the point of recognition, by the patient, to the lesion being correctly diagnosed was around 13.5 months.

**Conclusion:**

Earlier diagnosis of ALM requires education at both a patient and practitioner level.

## Background

Melanoma is a malignant tumour arising from melanocytes. The number of cases of the disease worldwide is increasing faster than any other form of cancer amongst Caucasians[1]. Although the disease is uncommon in the UK, the incidence of cutaneous melanoma continues to rise and it has been calculated that the lifetime risk for developing the disease is 1:120 for men and 1:95 for women[2]. Currently there are around 8500 new cases annually in the UK with around 1800 melanoma related deaths[3]. Australia has the highest annual incidence of melanoma in the world. The lifetime risk of developing melanoma before the age of 75 is 1: 24 for males and 1:34 for females. In 2003, there were 9,524 new cases of melanoma reported in Australia with an annual death rate of around 1500[4]. Cutaneous melanoma can develop at any site. The lower limb represents around 30% of all primary cutaneous melanomas, particularly in women, with the foot and ankle representing 3–15% of all cutaneous melanomas[5].

### Sub-types of Melanoma

Malignant melanoma (MM) is the commonest malignancy observed in the foot[6]. In 1969, Clark et al[7] histologically identified three sub-types – superficial spreading melanoma (SSM), nodular melanoma (NM) and lentigo maligna melanoma (LMM). In 1976, a fourth type, acral lentiginous melanoma (ALM) was added by Reed[8]. All sub-types of melanoma have been reported to arise on the foot with the exception of the LMM which occurs almost exclusively on the face[9].

### Acral Lentiginous Melanoma

The term ALM was first described by Reed[8] as a subtype of melanoma. It was so named because of its predilection of acral (distal) areas of the body, particularly the palms, soles and the sub-ungual areas, and its distinct radial or "lentiginous" growth phase. ALM represents the rarest of the four sub-types of cutaneous melanoma yet is the most common variety diagnosed on the foot[10]. Reed described its diagnosis as being based on its histological, intra-dermal features showing a diffuse proliferation of large atypical melanocytes along the epidermal-dermal junction which is dispersed in a lentiginous pattern with marked acanthosis and elongation of the rete ridges[8]. When reviewing terminology within the literature, confusion often arises with the use of the term "acral" with some papers describing "acral melanoma" which is merely an anatomical term for any sub-type of melanoma located on the palms, soles or sub-ungual region.

**Figure 1:**
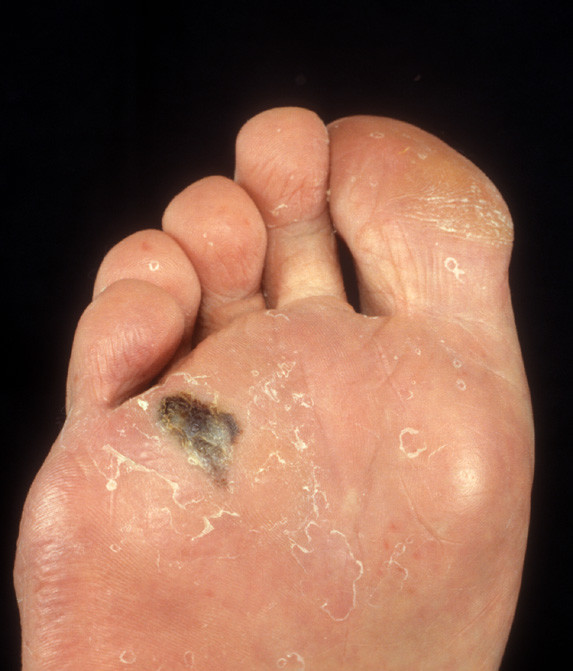
Acral lentiginous melanoma on the plantar surface.

ALM (figure 1) is the only sub-type of melanoma that occurs at the same rate in all races[11]. However, research data have demonstrated that melanomas in acral locations account for only around 1–7% of all cutaneous melanomas in Caucasians but has been shown to be significantly higher in Asian[12,13], Chinese[14,15], Japanese[16], Middle Eastern[17] and African populations[18,19]. This data reflects the low incidence of melanomas elsewhere on the body in the more pigmented skin types.

### Aetiology

As ALM occurs equally across all races, predominantly on an area that seldom receives much sun exposure it has been suggested that the aetiology is different to that of other sub-types of melanoma or that sun exposure is a lesser risk factor than melanoma elsewhere. Green et al[20] undertook a case control study of 275 melanomas diagnosed on the soles and palms to investigate risk factors. Interestingly, they found that sun exposure was a significant risk factor in the development of ALM despite their plantar and nail bed location. Furthermore, a high mole count on the soles and elsewhere on the body were associated risk factors (RR = 6.3 95% CI 2.5–15.6). Reinforcing this belief, other studies have demonstrated that increased sun exposure in an individual leads to the development of higher numbers of moles, especially in children[21].

Trauma as a cause has also been proposed as a possible risk factor for the development of ALM[20]. Penetrative injury of the foot showed significant association (RR = 5.0 CI 3.0–8.6) although the authors could not confirm from the data if the ALM actually developed at the original site of injury. In an earlier study, Briggs et al[22] reviewed a number of cases but suggested that incidental injury to the foot merely drew the patients attention to a pre-existing foot problem. Kaskel et al[23] suggested that trauma in acral areas such as the foot were to be expected more frequently and could find no evidence to identify trauma as an aetiology.

The prognosis of the disease, as with other sub-types of melanoma, is determined by the Breslow thickness of the lesion at diagnosis[24]. It has been suggested that ALM itself carries a worse prognosis than other melanoma – often as lesions are recognised later than melanoma on other body sites[25]. Following a number of cases late diagnosis occurring at a tertiary care centre, a study was set up to review cases of the disease in an attempt to identify common clinical factors.

## Methods

A database search was undertaken to identify all cases of ALM treated at the tertiary care melanoma centre located in a central London district. From these, notes were selected of patients presenting with a ALM (diagnosed by histology) on the ankle or below. In the period 2000 – 2006, twenty seven patients were identified and from their records clinical data including gender, age, ethnicity and diagnostic information were gathered and tabulated for review.

## Results

The cohort of patients totalled 27 (20 female and 7 male) with a female ratio of nearly 3:1. The patients' age at diagnosis ranged from 35 to 96 years. The average age of the patient at was 62.7 with no age difference between men and women (62.5 versus 62.8 respectively). The majority of patients reported their ethnicity as white (n = 16) in addition there were 7 Afro-Caribbean, 1 Chinese/Oriental and 3 unrecorded. Although not always recorded, patients had been reviewed at a number of other clinics with their lesions prior to reaching the dermatology department with a definitive diagnosis. These included a range of specialities – general practice (n = 5), podiatry/chiropody (n = 9), vascular clinics (n = 2), diabetology (n = 1) and plastic surgery (n = 1).

Nineteen of the lesions were reported on the right foot and eight on the left. All male patients exhibited ALM on their right foot only. The majority of lesions were located on the plantar surface (62%) with 2 on the ankle, 2 on the dorsum of the foot, 1 on the digit and 4 located in the nail bed (with 2 in the hallux and 2 in the fifth toe nail bed). One lesion site was stated simply as being on the "foot" (see table 1). Twenty-one (78%) of the lesions were reported as melanotic, three amelanotic (11%) and three (11%) were unknown.

**Table 1:** Summary of locations of ALM in 27 patients

**Location**	**Number**
**Plantar Surface**	**17**
Plantar Forefoot 6	6
(4 located under 1st met head.)	
Plantar Midfoot	5
Plantar Heel	6
	
**Dorsum of the foot**	**2**
**Ankle**	**2**
**Nail Bed**	**4**
(2 hallux, 2 fifth toe)	
	
**Digit (excluding nail unit)**	**1**
**Not Known**	**1**

**Total**	**27**

Data on the time from the patient first recognising something on their foot to diagnosis was available for 19 patients. The average time for women was 12.5 months versus 14.5 months in males. The most reported symptoms from patients were change in size and bleeding (see table 2). A number of lesions were misdiagnosed as warts (n = 4). Lesion thickness at diagnosis ranged from 0.84 mm to 13.30 mm. The mean thickness for women being 3.68 mm (n = 16) versus 4.41 mm in males (n = 6).

**Table 2:** Reported symptoms/diagnoses (21 patients). Reported symptom

**Symptoms**	**Number**
Change in Size	8
Bleeding	4
Change in colour	2
Change in form	2
Pain	1
Itching	1
	
**Previous Diagnoses**	
Wart	4
Fungal Infection	1
Haematoma	1
Ulcer	1

## Discussion

This set of patients represents a small cohort (n = 27) of a population from an urban area with a high ethnic mix. Interestingly, despite the wide ethnic diversity of the local area, a high proportion of this cohort were white (69%). Despite the wide spread of ages (35 – 96), the average age of the patient in this study was 62.6 years which concurs with similar studies[26,27] that ALM is most frequent in the 60–70 age group[25]. ALM appears to occur in an older age group, other types of melanoma having a peak incidence around 50 years of age, albeit with a wider age spread[2]. The female preponderance to ALM was 2.8:1 slightly higher than other published data [26-28] but still confirms that MM is a disease more common in females[3,10].

Within this study, the prime location for ALM was the plantar surface (65%), with 4 of these occurring under the first metatarsal head. A smaller number were seen in nail beds, ankle and dorsum of the foot. A similar prevalence pattern for the plantar area has been reported by Soon et al[27](61%) and Kuchelmeister[25] (65%) with sub-ungual lesions making up a smaller percentage of all cases of ALM. The four sub-ungual tumours in this study were located exclusively on the hallux (50%) and fifth toe (50%). The hallux has been consistently reported to be the most common area for sub-ungual lesions in the foot. Possible reasons for this are two-fold. Firstly, the hallux may be the most prevalent location owing to the larger proportion of nail tissue in this area. Secondly, one could debate the role of trauma. The hallux is typically an area of the forefoot more prone to abuse from footwear and one-off injury. In one case series from Germany, 6 patients with ALM reported tight footwear as a possible causative agent[23]. The authors went on to discuss that patients with acral melanoma tended to report a high rate of trauma compared to those with melanoma at other sites but this was not found to be statistically significant. Furthermore, one could hypothesize, if physical trauma was associated with melanoma, one would expect the foot show a more significant proportion of lesions on the foot as a result of the forces of weight bearing and locomotion.

Early recognition is the key to improving survival rates[29]. As cutaneous melanoma is a visible disease, both the patient and practitioner play a major role in recognising suspicious lesions. Initially, the time taken to reach a diagnosis depends on the patient's ability to recognise and seek professional advice. Secondly, diagnosis depends on the professional's capacity to recognise the lesion. Data were available for 19 patients showing that the time from first noticing a lesion to diagnosis ranged from 1 – 36 months, which shows similarities to other studies of patients with ALM[26]. Reasons for the delay were not examined in this study but have been reviewed by Richard et el[30]. In a series of 590 patients they examined the reasons for delay in melanoma diagnosis and discovered that male gender, increasing age and a low educational level were all risk factors for a later presentation to physicians. In a second paper[31] examining physician delays, acral locations and lack of lesion pigmentation were factors more likely to lead to a delay in diagnosis by a physician, particularly lesions in acral locations without pigmentation.

Within this study, symptoms or initial diagnoses were recorded for 21 patients. The most common reported symptom was a change in the size of the lesion (38%) followed by bleeding (19%), change in colour (9%) and change lesion form (becoming raised/nodular) (9%). Bleeding is a common feature in melanoma which have entered a vertical growth phase and have become ulcerated[2] and may represent a feature of advanced disease. The average lesion thickness in patients reporting bleeding was significantly higher in those not reporting it (mean thickness 6.13 mm v 3.8 mm) although due to the small numbers involved it was difficult to draw firm conclusions.

Seven of the twenty one lesions (33%) were initially misdiagnosed as other conditions (warts, a fungal infection, haematoma and an ulcer). Numerous papers have highlighted conditions including warts, tinea pedis, ulceration, infection, paronychia, haematoma, onychomycosis, ischaemic necrosis, pyogenic granuloma, ganglions and blisters which have been later discovered to be ALM [27,28,32-36]. Misdiagnosis is a common feature of melanoma on the foot but ALM in particular has been shown to be more likely mis-diagnosed than other sub-types of the disease[37]. Delays can in turn lead to a poorer prognosis for the patient. The misdiagnosis rate in this study was 33%, other have reported rates of between 33% – 67%[27,38].

It is appreciated that the results of this study represent a retrospective review of patient case notes which have some inherent bias – in particular that this data was collected at a tertiary centre where possibly only more complex cases are seen. However, in view of the relative rarity of the condition, twenty-six cases represent a sizeable cohort, which has been shown to be concurrent when compared to literature on this topic.

This paper has highlighted an uncommon but serious lesion which may present for the first time to Chiropodists and Podiatrists. One third of the lesions, in the presented cohort, were seen prior to diagnosis by a chiropodist or podiatrist. Unfortunately, typical features of melanoma as exhibited by the "ABCDE" rule may not be present in a proportion of ALM and so misdiagnosis remains a significant risk. Therefore it is important to remain vigilant and where there is clinical suspicion, patients should be referred for a prompt dermatological opinion. In suggesting ways to heighten awareness, the typical patient profile should be borne in mind as well as continuing the patient health education message. In addition, dermoscopy has been demonstrated as a useful, non-invasive technique to increase sensitivity in acral lesions[39]improving early recognition.

## Conclusion

Acral lentigious melanoma is an uncommon malignant tumour which can occur on the foot. This study provided clinical data from 27 cases based on a mainly white, urban population. A third of cases in this series were misdiagnosed before reaching the skin clinic with a proportion of patients having been seen by a number of specialities prior to diagnosis. Lesions were most common on the plantar surface (62%). The average time from patients first noticing something to diagnosis was 13.5 months. The most common reported symptoms were a enlargement of the lesion (38%) and bleeding (19%). Further studies are required to better understand the aetiology and pathology of this unusual but serious tumour.

## Competing interests

The authors declare that they have no competing interests.

## Authors' contributions

Please see sample text in the instructions for authors.
